# The Second Annual Report of the Council

**Published:** 1917-07-07

**Authors:** 


### THE COLLEGE OF NURSING, LIMITED.

### The Second Annual Report of the Council.

In presenting their second Annual Report to the General Meeting the Council desires to give some account of the progress already made by the College, and some indication of the questions which may be expected to demand attention in the coming year. If much has already been done, more still remains to do, and it is to its members throughout the country that the College looks to make its aims and objects more widely known and appreciated.

### The Progress of the Register.

2. As may be remembered, no serious efforts were made to enrol members until after the first Annual Meeting in August 1916, when certain alterations in the Constitution were made, which have now been incorporated in the revised Articles of Association. The propaganda of Registration began immediately afterwards, and has been successful beyond all expectation. Up to the end of last month there have been received more than 7,000 applications for membership, about ten per cent. of which were from nurses whose record of training did not comply with the standards laid down by the Council. It is satisfactory to note that the College has attracted members from every part of the British Empire, whilst the large number of applications from nurses on active service, emphasising, as it does, the need felt even at such a time as this for the better organisation of the profession on the lines laid down by the College, is full of encouragement for those at home who, busy as they are with war work, have to find time to cope with the steadily increasing demands which Council and Committee meetings make upon their attention and energies. Much of the success of the Registration Campaign is undoubtedly due to the efforts of the rank and file, who, by personal canvass, have stimulated an interest in their fellow nurses, and whose efforts have been seconded by the wide publicity afforded to the College and its policy in all sections of the Nursing Press.

### Over Centralisation Avoided.

3. The disadvantages of excessive centralisation have been present to the minds of the Council from the first, and, acting under the powers given them in the Memorandum of Association (para. 79, 80), they established in September 1916 a Scottish Board in accordance with the recommendations laid before them by a meeting in Edinburgh, held in May, of representatives of the leading hospitals and infirmaries in Scotland. In February of the present year an Irish Board was established, with a similar Constitution. The *personnel* of these Boards gives the strongest assurance that the interests of Scotland and Ireland will not be overlooked, and that local differences and difficulties will be considered sympathetically on the spot by those familiar with the conditions. At the same time, the Central Administration has been strengthened by the inclusion on the Council of Scottish and Irish representatives, nominated by their respective Boards, who together form nearly one-third of the total membership of the Council. Important work has already been done by the Scottish Board, which, having provided itself with an office and secretary in Edinburgh, has been engaged in considering and advising upon the applications for membership received from nurses trained in that country. The Irish Board is busy with similar work for Ireland. The Annual Reports of both Boards are circulated herewith.

### THE COLLEGE OF NURSING, Ltd.—(*continued*).

THE CONCERT AT QUEEN'S HALL.

4. Mention should be made, with sincere appreciation and thanks, of the concert given last May, by Mr. Victor Beigel, at the Queen's Hall, to which upwards of 2,000 nurses were invited by the Council. Both H.M. Queen Alexandra and H.R.H. Princess Christian honoured the occasion by their presence, and thus showed once more the keen interest they take in all that makes for the good of the nursing profession.

THE COUNCIL'S SUCCESSFUL ACTION.

5. The Council may, perhaps, refer with some satisfaction to the success which attended their efforts to obtain due representation for the nursing profession on the very important Committee appointed by the Secretary of State for War last September, to consider "the existing system of obtaining nurses for the hospitals for sick and wounded soldiers at home and abroad, and to make such recommendations as they may consider necessary for augmenting the supply." The Report of this Committee has recently been made public, and it is perhaps not unreasonable to attribute some of the general approval extended to its findings to the fact that upon it, in addition to the two Matrons-in-Chief, place was found for seven members of the College Council, who from their wide knowledge of the circumstances of Civil and Military Nursing were in a position to tender to the authorities of the War Office very sound and valuable advice.

STATE REGISTRATION AND A HOPE FOR UNION.

6. Regarding the State Registration of Nurses as the most important matter in the field of general policy, the Council has to record with sincere regret the fact that, up to the present, they have been unable to arrive at an understanding with the Central Committee for the State Registration of Nurses so as jointly to promote an agreed Bill. At a time when substantial progress seemed to have been made, negotiations were broken off by the Central Committee upon grounds which appeared to many to be totally inadequate, the more so as a short Bill, confined to essentials and leaving details, although important, to be settled with the Privy Council, would, it is believed, have had a good chance of passing through Parliament as a War Measure, even in the present Session. There is, however, still hope that wiser counsels may prevail. Here and now it is unnecessary to say more, since the points of difference between the College Bill and the Bill of the Central Committee have been fully set out in a memorandum lately circulated to members.

THE COLLEGE PROPAGANDA.

7. A certain amount of propaganda work has been done by holding meetings in England, Wales, Scotland, and Ireland in support of the College, and every meeting has been attended with very satisfactory results, many of those present showing themselves ready to co-operate in furthering the objects the College has in view as regards the higher education and improved training of nurses. In some schools conditions have already been altered in conformity with the advice of the Council. The thanks of all are due to the members of the Council, and others, who, often at great personal inconvenience, have thrown themselves into this most important pioneer work. We hope and expect that many other members of the College will volunteer their services so as to consolidate the positions thus won by those who have led the advance.

WORK OF AMALGAMATION NEARLY COMPLETED.

8. Recognising the identity of many of the aims of the Royal British Nurses' Association with those of the College, and the advantages which would accrue to the latter body by associating itself with a Corporation already possessing a Royal Charter, the Council early entered into negotiations with the Council of the Royal British Nurses' Association with a view to amalgamation under the title of the "Royal British College of Nursing." An agreement for this purpose, to which was appended a Supplemental Charter and new bye-laws, was adopted by the Council in October 1916, and was approved *nem. con.* by the members of the Royal British Nurses' Association at a General Meeting held in January 1917. The New Charter is now before the Privy Council, and a Petition against it has been lodged by the Society for the State Registration of Trained Nurses. It is probable that a decision will not long be delayed, and, should it prove to be favourable, the two bodies will be in a strong position when, as is incumbent on them under the Agreement, they take immediate steps to obtain from Parliament an Act for the Registration of Trained Nurses. The first official Register under the Bill will be identical with the Joint Roll of Membership of the Royal British Nurses' Association and the College of Nursing, Ltd.

CLAIMS OF POOR-LAW ASSOCIATIONS AND NURSES.

9. The Council has devoted anxious consideration to the claims of various Poor-Law Associations on behalf of nurses trained in Poor-Law institutions. It is certain that no scheme for the State Registration of Nurses would be fair unless a place were found in it, on equitable terms, for those who constitute so large and important a section of the nursing profession. The Council is fortunate in having already on it matrons and others, who may claim to speak with knowledge and authority for this branch of the profession, and it may become advisable to strengthen it still further on this side of its activities.

A REVIEW OF THE YEAR'S WORK.

10. From a review of the work already done during the past year, and from the encouragement already extended to its efforts, the Council feels that the College may face the future hopefully. The work of registration so well begun has to be continued unremittingly, and all members are earnestly invited to co-operate in it by impressing on their friends the paramount importance of securing better organisation for the profession, protection for its members, and the improvement of its status and economic condition. In the coming year, so far as the preoccupations of the war will permit, attention will be directed to the difficult questions which arise when it is sought to translate into practical regulations and detailed syllabuses the ideals contained in such phrases as the uniform curriculum and the one-portal system of examination. Moreover, if a supplemental Charter is granted, the College as a "Company limited by guarantee" will cease to exist, and the new Royal British College of Nursing will take its place; a scheme will have to be formulated and laid before the Privy Council to provide for the due representation of the nurses on the first elected Council of Royal College; and the Registration Bill first promoted by the College will require careful revision in the light of experience and of altered condi-

